# Structure, Process, and Mortality Associated with Acute Coronary Syndrome Management in Guatemala’s National Healthcare System: The ACS-GT Registry

**DOI:** 10.5334/gh.1168

**Published:** 2022-12-01

**Authors:** José Antonio Cornejo-Guerra, Magda Isabel Ramos-Castro, Mariana Gil-Salazar, Sofia Leal-Wittkowsky, Juan Carlos Santis-Mejía, Elisa María Anleu-De León, Oscar Fernando Castro-Alvarado, Boris Rudy Alexander López-Quiñónez, Edgar Alexander Illescas-González, Paola Overall-Salazar, Luis Antonio Rodríguez-Cifuentes, Karla Yesenia Miranda-Sandoval, Juan Pablo Pineda, Kevin Oneal Flores-Andrade, Roberto Antonio Pérez-Reyes, Sofía Waleska Girón-Blas, Josué Fernando Samayoa-Ruano

**Affiliations:** 1 Universidad de San Carlos de Guatemala, Guatemala; 2 Interventional Cardiology Department. Instituto Nacional de Cardiología Ignacio Chávez, México; 3 Universidad Nacional Autónoma de México, México; 4 Internal Medicine Department. Hospital General San Juan de Dios, Guatemala; 5 Universidad Francisco Marroquín, Guatemala; 6 Institute of Nutrition and Food Technology (INTA), University of Chile, Santiago, Chile; 7 Centro Universitario de Occidente de la Universidad de San Carlos de Guatemala, Guatemala; 8 Hospital Regional de Occidente, Guatemala; 9 Hospital Departamental de Totonicapán, Guatemala; 10 Hospital Nacional Pedro de Bethancourt, Guatemala; 11 Hospital Regional de San Benito Petén, Guatemala; 12 Hospital Regional de Cobán, Guatemala; 13 Hospital Regional de Zacapa, Guatemala

**Keywords:** acute coronary syndrome, Guatemala, ST elevation myocardial infarction, non-ST elevated myocardial infarction, fibrinolysis, percutaneous coronary intervention

## Abstract

**Background:**

Acute coronary syndromes (ACS) include ST-segment elevation myocardial infarction (STEMI), non-ST-segment elevation myocardial infarction (NSTEMI), and unstable angina (UA). The leading cause of mortality in Guatemala is acute myocardial infarction (AMI) and there is no established national policy nor current standard of care.

**Objective:**

Describe the factors that influence ACS outcome, evaluating the national healthcare system’s quality of care based on the Donabedian health model.

**Methods:**

The ACS-Gt study is an observational, multicentre, and prospective national registry. A total of 109 ACS adult patients admitted at six hospitals from Guatemala’s National Healthcare System were included. These represent six out of the country’s eight geographic regions. Data enrolment took place from February 2020 to January 2021. Data was assessed using chi-square test, Student’s t-test, or Mann-Whitney U test, whichever applied. A p-value < 0.05 was considered statistically significant.

**Results:**

One hundred and nine patients met inclusion criteria (80.7% STEMI, 19.3% NSTEMI/UA). The population was predominantly male, (68%) hypertensive (49.5%), and diabetic (45.9%). Fifty-nine percent of STEMI patients received fibrinolysis (alteplase 65.4%) and none for primary Percutaneous Coronary Intervention (pPCI). Reperfusion success rate was 65%, and none were taken to PCI afterwards in the recommended time period (2–24 hours). Prognostic delays in STEMI were significantly prolonged in comparison with European guidelines goals. Optimal in-hospital medical therapy was 8.3%, and in-hospital mortality was 20.4%.

**Conclusions:**

There is poor access to ACS pharmacological treatment, low reperfusion rate, and no primary, urgent, or rescue PCI available. No patient fulfilled the recommended time period between successful fibrinolysis and PCI. Resources are limited and inefficiently used.

## Background

Acute coronary syndromes (ACS) include ST-segment elevation myocardial infarction (STEMI), non-ST segment elevation myocardial infarction (NSTEMI), and unstable angina (UA) [1]. Globally, ischaemic heart disease was the leading cause of death and the second leading cause of disability-adjusted life years in 2019. World Health Organization indicators determined the leading cause of mortality in Guatemala to be ischaemic heart disease [2]. Currently, there is no established standard of care for ACS in Guatemala [3].

ACCESS (Acute Coronary Event Strategies Survey), an international observational registry, determined STEMI to be the most prevalent type of ACS in Guatemala. The ACCESS registry was the first to provide relevant information about acute myocardial infarction (AMI) management in Guatemala. However, it only included two public and a few private health centres located in Guatemala City. It provided evidence that no patients received primary Percutaneous Coronary Intervention (pPCI), and only 12% received elective PCI [3].

STEMI mortality is directly dependent on early reperfusion therapy, which in turn, relies on the structure and process of the healthcare system. Early administration of fibrinolytic therapy has proven to reduce the size of the infarct, preserve ventricular function and reduce mortality [4]. This is true 10 years after the event, particularly in patients that received fibrinolysis three hours after symptom onset [5].

In Guatemala, pharmacological reperfusion is the main method available given the lack of access to PCI centres [6]. When fibrinolysis is administered less than two hours after symptom onset, mortality reduction can be greater than with PCI [4]. Therefore, timely administration of fibrinolytic therapy is crucial. A meta-analysis demonstrated that the benefit of thrombolytic therapy initiated within 60–90 minutes after onset of symptoms can be estimated at 60–80 additional patients alive at 1 month per 1000 treated with conventional therapy [7]. Despite substantial evidence of the effectiveness of fibrinolytic therapy, its use remains low. In the PRIAMHO study, fibrinolytic use was 41% and in the GESIR-5 study 35% [8,9].

There are various prognostic time intervals measured from symptom onset to reperfusion, which are useful in evaluating the efficacy of the health care system: first medical contact, door-in to door-out, door-balloon, door-needle, and total ischaemic time. By measuring and analysing these delays, it is possible to identify the step in which the treatment system is deficient.

Direct comparison between fibrinolysis and PCI is controversial. European Society of Cardiology (ESC) guidelines for STEMI management recommend pPCI as the preferred reperfusion strategy within indicated timeframes [4]. Performing pPCI requires a coordinated interdisciplinary effort including adequate transport and availability of experienced health professionals and resources. If deficiencies of the structure and system of care are known, it will be possible to establish adequate protocols and decentralize treatment for timely reperfusion and improvement of patient prognosis.

This registry was created in order to collect ACS data representative of the national health system. It pretends to describe the process, structure, and outcome of ACS care in Guatemala based on the Donabedian health model [10].

## Objectives

Describe the main factors that influence patients’ ACS health outcome in Guatemala. Determine demographic and clinical characteristics among study subjects and outline the process and structure of the national healthcare care system.

## Methods

### Study design and population

The methodology has been previously described [11]. Briefly, the ACS-Gt study is an observational, multicentre, prospective registry that included adult patients with ACS admitted at five regional second level hospitals and one tertiary hospital part of Guatemala’s National Healthcare System. These represent six out of the country’s eight geographic regions. The National Ministry of Public Health is a healthcare provider for nearly 75% of the population [6].

Patients with signs, symptoms, electrocardiogram findings, and cardiac biomarkers compatible with ACS were eligible for enrolment from February 2020 to January 2021. Out of 112 collected patients, 109 met inclusion criteria. This study was approved by the Bioethics Committee in Health Investigation of Universidad de San Carlos de Guatemala (a public higher-education institution).

### Data collection

**Figure 1:**
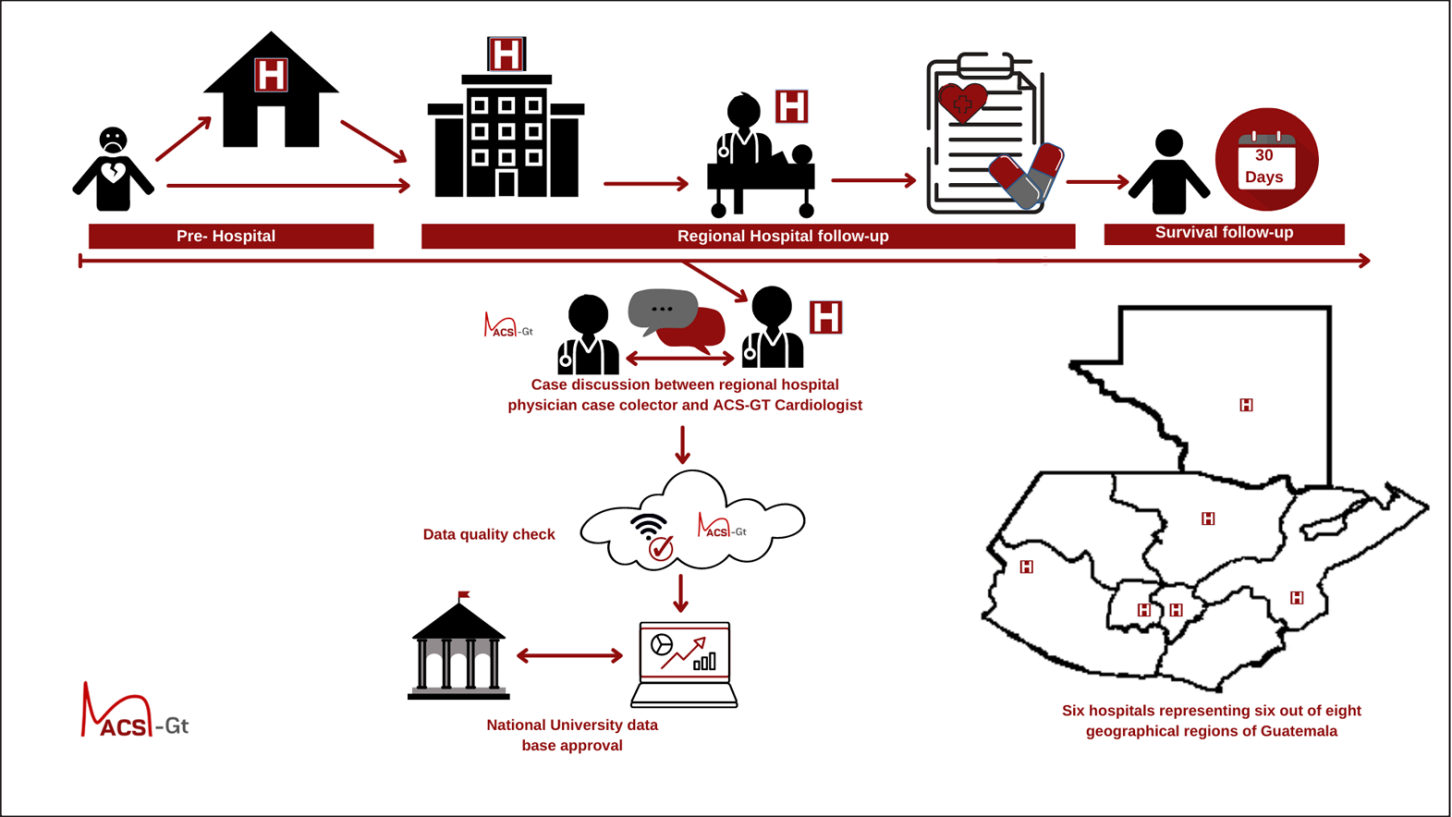
ACS-Gt Registry Methodology.

Data was collected by trained physicians working at six designated hospitals. All investigators underwent training in bioethics, data collection, and received a standardized manual with operational definitions. The survey was filled out using the electronic database system REDCap (Research Electronic Data Capture) composed of sociodemographic questions and those that described the process, structure, and outcome of the healthcare system of ACS (Figure 1).

The survey was completed in three stages. The first was conducted at hospital admission and composed of baseline characteristics, traditional risk factors, clinical presentation, electrocardiogram (ECG) findings, laboratory data, risk stratification, and reperfusion therapy. The second was conducted during hospital stay, composed of follow-ups related to non-cardiovascular and cardiovascular outcomes. At hospital discharge, treatment, left ventricular ejection fraction (LVEF), and future appointments were taken into account. Finally, a follow-up 30 days after the event was assessed to evaluate vital status. All patients were given an informed consent form for participation and follow-up approval.

Collected data underwent a weekly review by at least three physicians from the research team. Data integrity was verified according to inclusion criteria and correction of discrepancies to ensure the collection of high-quality data. All cases with a debatable diagnosis were analysed by members of the scientific committee composed of cardiologists and internal medicine physicians.

### Variable definition

The research team combined international ACS guidelines and Guatemala’s healthcare context to provide standardized variable definitions (supplement Table 1).

STEMI was considered when a patient with ACS symptoms had persistent ST-segment elevation in at least two contiguous leads (≥2.5 mm in men <40 years, ≥2 mm in men ≥40 years, ≥1.5 mm in women in leads V_2_–V_3_ and/or ≥1 mm in the other leads, or ≥0.5 mm in leads V_7_–V_9_) [4]. Diagnosis was confirmed with abnormality of cardiac enzymes. The presence of a right bundle branch block with ST-segment elevation or left bundle branch block that met Sgarbossa criteria was also considered STEMI [4,12,13]. NSTEMI/UA criteria are detailed in supplement Table 1. Failed thrombolysis was defined as ST-segment descent failure of at least 50% in the most prominent lead, persistence of angina, and/or worsening of hemodynamic parameters 90 minutes after thrombolysis [14,15].

Optimal medical therapy (OMT) at admission was defined by the combined prescription of aspirin, P2Y12 inhibitors, heparin or enoxaparin, and fibrinolytic therapy according to 2017 STEMI and 2015 NSTEMI European guidelines [4,16]. OMT at discharge (DOMT) was considered with prescription of aspirin, P2Y12 inhibitors, and statins. Prescription of angiotensin-converting enzyme (ACE) or inhibitors/angiotensin-receptor blockers (ARB), β-blockers, or angiotensin receptor-neprilysin inhibitor (ARNI) in patients with reduced left ventricular ejection fraction (rLVEF) (≤40%), was also regarded as DOMT.

### Data analysis

SPSS.24 system was used for data analysis. Continuous variables were expressed as mean and standard deviations (± SD) or medians and interquartile ranges. Categorical variables were described as frequency and percentages. Statistical tests, Chi-square, Student’s t-test, or Mann-Whitney U test were performed, whichever applied. A p-value < 0.05 was considered statistically significant.

## Results

The registry collected 109 patients with ACS; mean age was 61.6 years and the majority were male (68%). Almost 45% lived in rural areas, most of them had elementary school education level and worked in informal commerce. No difference was found between demographic variables in STEMI and NSTEMI/UA patients, although NSTEMI/UA patients were older. Most STEMI and NSTEMI/UA patients had a past medical history of systemic hypertension and diabetes mellitus. However, more STEMI patients smoked and more NSTEMI/UA had history of an ACS event. While previously used medications were similar between STEMI and NSTEMI/UA, the latter had higher use of ACEI/ARB, aspirin, statins, and insulin (Table 1).

**Table 1:** Demographic and clinical characteristics.


	STEMI	NSTEMI/UA	TOTAL	P

	N = 88	N = 21	N = 109	

	x̄ ± SD	x̄ ± SD	x̄ ± SD	

Age ∼ years	60.9 ± 11.2	64.9 ± 12.6	61.6 ± 11.6	0.15

Weight ∼ kg	72.7 ± 10.4	70.6 ± 12.3	70.8 ± 14	0.43

	**N (%)**	**N (%)**	**N (%)**	

**Gender**				0.25

Male	69 (78)	14 (66.7)	83 (68)	

**Housing**				0.98

Rural	38 (43.2)	9 (42.9)	47 (43.1)	

**Educational level**				0.43

Illiterate	11 (12.6)	4 (19)	15 (13.9)	

Elementary school	37 (42.5)	12 (57.1)	49 (45.4)	

High school	25 (28.7)	2 (9.5)	27 (25)	

Technical degree	10 (11.5)	2 (9.5)	12 (11.1)	

Bachelor’s degree or higher	4 (4.6)	1 (4.8)	5 (4.6)	

**Occupation**				0.41

Informal commerce	19 (21.8)	8 (38.1)	27 (25)	

Housewife	16 (18.4)	5 (23.8)	21 (19.4)	

Unemployed	18 (20.7)	1 (4.8)	19 (17.6)	

Technical	14 (16.1)	3 (14.3)	17 (15.7)	

Professional	8 (9.2)	2 (9.5)	10 (9.3)	

Agriculture	6 (6.9)	2 (9.5)	8 (7.4)	

Formal commerce	6 (6.9)	0.0	6 (5.6)	

**Established hospital protocol for acute coronary syndrome**	11 (12.5)	1 (4.8)	12 (11)	0.31

**Medical history**				

Systemic hypertension	43 (48.9)	11 (52.4)	54 (49.5)	0.77

Diabetes mellitus	40 (45.5)	10 (47.6)	50 (45.9)	0.85

Smoking	24 (27.3)	3 (14.3)	27 (24.8)	0.22

Dyslipidaemia	19 (21.8)	2 (9.5)	21 (19.4)	0.2

Acute coronary syndrome	11 (12.6)	4 (19)	15 (13.9)	0.44

Other	20 (22)	6 (21)	26 (23)	0.62

**Medication history**				

ACEI/ARB	34 (39.1)	9 (42.9)	43 (39.8)	0.75

Acetylsalicylic acid	7 (8)	5 (23.8)	12 (11.1)	0.03

Insulin	9 (10.3)	3 (14.3)	12 (11.1)	0.6

CCB	5 (5.7)	4 (19)	9 (8.3)	0.04

P2Y_12_ inhibitor	5 (5.7)	1 (4.8)	6 (5.6)	0.86

β-blocker	5 (5.7)	1 (4.8)	6 (5.6)	0.86

Statins	3 (3.4)	2 (9.5)	5 (4.6)	0.24

Nitrites	1 (1.1)	1 (4.8)	2 (1.9)	0.27


STEMI: ST-elevation myocardial infarction, NSTEMI: non-ST elevation myocardial infarction, ACEI: Angiotensin-converting enzyme inhibitors, ARB: Angiotensin II receptor blocker, CCB: Calcium channel blocker.

Most STEMI patients had an anterior myocardial infarction, only 59% received fibrinolysis (no pPCI was performed due to lack of public-PCI centres). Alteplase was the most commonly used fibrinolytic, mostly at a third level hospital. Regional hospitals used Streptokinase. Fibrinolysis was not performed in 50% of patients due to late presentation (>12 hours from symptom onset) and in 36% of patients due to lack of consideration by physician (error in clinical scenario or unskilled ECG interpretation). Fibrinolysis was mostly performed at a public hospital, however, 13.5% was performed at a private hospital by patient’s out-of-pocket expense. Fibrinolysis was considered successful in 65% of patients but none were taken to PCI after the procedure (pharmacoinvasive strategy) during the recommended time period. One rescue PCI was performed at a private hospital. Nearly 20% of non-reperfused patients underwent angiography or PCI, nevertheless, only 57% had an ischaemia or viability test completed. Patients with NSTEMI/UA rarely had angiography or PCI performed, and most were diagnosed with multivessel disease (Table 2).

**Table 2:** STEMI and NSTEMI individual characteristics.


STEMI CHARACTERISTICS	STEMI

	N = 88

	N (%)

**Electrocardiographic location**	

Anterior	45 (51.1)

Inferior	38 (43.2)

Lateral	5 (5.7)

**Reperfusion**	52 (59.1)

**Type of reperfusion**	

Fibrinolysis	52 (100)

**Fibrinolytic**	

Alteplase	34 (65.4)

Streptokinase	17 (32.7)

Tenecteplase	1 (1.9) *

**Reason PCI was not performed**	

No cardiac catherization laboratory	65 (73.9)

Time from symptom onset > 12 hours	13 (14.8)

Not considered by physician	7 (8)

Patient refused treatment	2 (2.3)

Hospital transfer not accepted	1 (1.1)

**Reason fibrinolysis was not performed**	

Time from symptom onset > 12 hours	18 (50)

Not considered by physician	13 (36.1)

Lack of medical supplies	3 (8.3)

Contraindicated	1 (2.8)

Patient refused treatment	1 (2.8)

**Angiography/PCI use in non-reperfused myocardial infarction**	7 (19.4)

**Ischaemia or viability evaluated prior to angiography/PCI in non-reperfused myocardial infarction**	4 (57)

**Treatment centre where fibrinolysis was performed**	

Public hospital (MSPAS)	44 (84.6)

Private hospital	7 (13.5)

Guatemalan social security institute (IGSS)	1 (1.9)

**Successful fibrinolysis**	34 (65.4)

**Angiography/PCI after successful fibrinolysis**	17 (50)

**Within first 24 hours**	0.0

**After 24 hours**	17 (100)

**Cause of failed fibrinolysis**	

Both	6 (50)

Persistent ischaemia	5 (41.7)

Failure of ST segment descent	1 (8.3)

**Rescue angioplasty**	1 (14.3) *

**NSTEMI/UA CHARACTERISTICS**	**NSTEMI/UA**

	**N = 21**

	**N (%)**

**Angiography/PCI performed**	5 (23.8)

Diagnostic	4 (80)

PCI	1 (20)

	**X̄± SD**

Days until angiography/PCI was performed	6.4 ± 2.8

**Electrocardiographic findings at admission**	

T-wave inversion	5 (29.4)

ST-segment depression	5 (29.4)

No alterations	4 (23.5)

ST-depression in more than 6 leads and ST-elevation in aVR	2 (11.8)

Left Bundle Branch Block	1 (5.9)

**Crusade**	35.8 ± 20.8


* Performed at a private hospital (after the procedure patient was transferred back for treatment at public hospital). STEMI: ST-elevation myocardial infarction, NSTEMI: non-ST elevation myocardial infarction, PCI: percutaneous coronary intervention, MSPAS: “*Ministerio de Salud Pública y Asistencia Social*” (ministry of public health and social assistance), IGSS: “*Instituto Guatemalteco de Seguridad social*” (Guatemalan social security institute), UA: unstable angina.

Clinical presentation between study groups was similar. However, GRACE score was significantly higher in the NSTEMI/UA group, whereas TIMI score was higher in STEMI. Electrocardiographic findings demonstrated that most third-degree AV block patients had STEMI and all patients with pacemaker rhythm had a NSTEMI/UA (Table 3). Biochemical characteristics at hospital admission can be found in supplement Table 2.

**Table 3:** Clinical Presentation.


	STEMI	NSTEMI/UA	TOTAL	P

	N = 88	N = 21	N = 109	

	x̄ ± SD	x̄ ± SD	x̄ ± SD	

Heart rate ∼ Bpm	80.3 ± 24.9	91.4 ± 31.7	82.4 ± 26.5	0.08

GRACE	129 ± 32	109 ± 32	125 ± 32.7	0.01

	**MEDIAN (25–75)**	**MEDIAN (25–75)**	**MEDIAN (25–75)**	

Systolic blood pressure ∼ mmHg	115 (91.2–134.7)	130 (110–140)	120 (100–139)	0.06

Oxygen saturation ∼ **%**	95 (91.7–97)	92 (86–96)	94 (90.2–97)	0.07

Diastolic blood pressure ∼ mmHg	70 (60–84.5)	80 (70–86)	70 (60–84)	0.22

Temperature ∼ °C	37 (36.5–37)	37 (36.7–37)	37 (36.5–37)	0.36

Respiratory rate ∼ Rpm	18 (16–22)	20 (17–24)	19 (16–22)	0.23

	**N (%)**	**N (%)**	**N (%)**	

Typical angina	76 (86.4)	16 (76.2)	92 (84.4)	0.24

Dyspnoea	18 (20.5)	9 (42.9)	27 (24.8)	0.03

Atypical angina	8 (9.1)	1 (4.8)	9 (8.3)	0.51

Syncope	8 (9.1)	1 (4.8)	9 (8.3)	0.51

Cardiac arrest	1 (1.1)	0.0	1 (0.9)	0.62

**Electrocardiographic findings at admission**				

Sinus rhythm	66 (75)	19 (90.5)	85 (78)	0.12

Third-degree AV block	14 (15.9)	0.0	14 (12.8)	0.05

Ventricular extrasystoles	4 (4.5)	1 (4.8)	5 (4.6)	0.96

Second-degree AV block	4 (4.5)	0.0	4 (3.7)	0.32

Other	2 (2.3)	1 (4.8)	3 (2.8)	0.53

Pacemaker rhythm	0.0	2 (9.5)	2 (1.8)	<0.01

Atrial fibrillation	1 (1.1)	0.0	1 (0.9)	0.62

First-degree AV block	1 (1.1)	0.0	1 (0.9)	0.62

**Killip-Kimball**				0.04

I	43 (48.9)	13 (61.9)	56 (51.4)	

II	27 (30.7)	4 (19)	31 (28.4)	

III	5 (5.7)	4 (19)	9 (8.3)	

IV	13 (14.8)	0.0	13 (11.9)	

**TIMI**				<0.01

0	0.0	2 (9.5)	2 (1.8)	

1	3 (3.4)	2 (9.5)	5 (4.6)	

2	8 (9.1)	6 (28.6)	14 (12.8)	

3	11 (12.5)	8 (38)	19 (17.4)	

4	9 (10)	2 (9.5)	11 (10)	

5	12 (13.6)	0.0	12 (11)	

6	8 (9.1)	1 (4.8)	9 (8.3)	

≥7	37 (42)	0.0	37 (34)	


STEMI: ST-elevation myocardial infarction, NSTEMI: non-ST elevation myocardial infarction, UA: unstable angina, Bpm: beats per minute, GRACE: Grace Risk Score, Rpm: respirations per minute, mmHg: millimetres of mercury, °C: degrees Celsius, AV: atrioventricular, Killip-Kimball: prognostic score in acute coronary syndrome evaluating the risk of death during the first 30 days, TIMI: score that assesses mortality, reinfarction or recurrent ischaemia during the first 14 days.

As mentioned previously, there were various points in the ACS system of care measured. These were divided into two main categories: prehospital and intrahospital care. Within prehospital delay, first medical contact (FMC) was longer in STEMI. One-third of ambulances were provided by the Public National Health System, and the rest by patient’s expense or the Social Security Institute. For the most part, transfer destination was a public hospital, but 13% of STEMI patients were transferred to a private hospital. The most common reason for a second transfer in STEMI was for fibrinolysis and in NSTEMI/UA elective PCI. Transfer conditions were suboptimal (Table 4).

**Table 4:** Process and delay.


	STEMI	NSTEMI/UA	TOTAL	P

	N = 88	N = 21	N = 109	

	x̄ ± SD	x̄ ± SD	x̄ ± SD	

**Prehospital delay**				

Time between ambulance call and arrival ∼ minutes*	18.4 ± 14.6	30 ± 23.7	20.7 ± 16.8	0.17

	**MEDIAN (25–75)**	**MEDIAN (25–75)**	**MEDIAN (25–75)**	

Transfer time to FMC ∼ minutes	22.5 (20–38.7)	30 (12.5–60)	25 (17.5–42.5)	0.56

FMC ∼ minutes	300 (126.2–842.2)	185 (75–1035)	180 (74–465)	0.37

Transfer time from FMC to final treatment centre ∼minutes	30 (20–60)	120 (10–180)	80 (30–141)	0.46

	**N (%)**	**N (%)**	**N (%)**	**P**

**Ambulance provided by**				0.96

Public hospital (MSPAS)	11 (37.9)	1 (33.3)	12 (37.5)	

Patient expense	8 (27.6)	1 (33.3)	9 (28.1)	

Social security institute (IGSS)	8 (27.6)	1 (33.3)	9 (28.1)	

Patient transport	2 (6.9)	0.0	2 (6.3)	

**Transfer destination**				

Public hospital (MSPAS)	24 (82.8)	3 (100)	27 (84.4)	

Private hospital**	4 (13.8)	0.0	4 (12.5)	

Social security institute (IGSS)	1 (3.4)	0.0	1 (3.1)	

**Reason for second transfer**				<0.01

Elective PCI	8 (27.6)	1 (33.3)	9 (28.1)	

Family request	3 (10.3)	0.0	3 (9.4)	

Intensive care	2 (6.9)	0.0	2 (6.3)	

Lack of physical space	0.0	1 (33.3)	1 (3.1)	

Diagnostic approach	0.0	1 (33.3)	1 (3.1)	

Fibrinolysis	11 (37.9)	–	–	

Pharmaco-invasive strategy (after successful fibrinolysis)	3 (10.3)	–	–	

Rescue PCI	2 (6.9)	–	–	

**Transfer characteristics**				

Performed by paramedic	14 (56)	2 (66.7)	16 (57.1)	0.9

Ambulance oxygen tank	15 (65.2)	1 (33.3)	16 (61.5)	0.34

Ambulance heart monitor	8 (34.8)	0.0	8 (30.8)	0.34

Ambulance defibrillator	5 (21.7)	0.0	5 (19.2)	0.53


* Only applies if the patient was transferred by ambulance. ** Only applies to patients who were evaluated or treated at a private hospital and later referred to a public hospital. Patients who only received treatment at a private hospital were not included. STEMI: ST-elevation myocardial infarction, NSTEMI: non-ST elevation myocardial infarction, UA: unstable angina, FMC: first medical contact, PCI: percutaneous coronary intervention, MSPAS: “*Ministerio de Salud Pública y Asistencia Social*” (ministry of public health and social assistance), IGSS: “*Instituto Guatemalteco de Seguridad social*” (Guatemalan social security institute).

Prognostic delays in STEMI were measured in order to understand the process and structure of the current health system. We compared time intervals recommended by ESC guidelines with ACS-Gt results (Table 5). Within intrahospital delay, all intervals were significantly prolonged in comparison with ESC guidelines, except the time from ECG to STEMI diagnosis.

**Table 5:** STEMI–delay in definitive treatment: ACS-Gt registry vs. ESC guidelines.


	ACS-GT	ESC-STEMI	DIFFERENCE	P

	MEDIAN (25–75)			

Time between ECG and STEMI diagnosis ∼ minutes	10 (5-20)	<10	0.0	0.44

Door to needle ∼ minutes	52.5 (27.7-71)	<10	+ 42	<0.01

Door-in to door-out ∼ minutes	120 (35-285)	<30	+ 90	<0.01

Total ischaemic time ∼ minutes	500 (388-720)	<120	+ 380	<0.01

	**X̄ ± SD**			

Total ischaemic time (patients presenting <12 h) ∼ minutes	439.5 ± 139.6	<120	+ 319	<0.01

Time in which Angiography/PCI was performed after successful fibrinolysis ∼ hours*	205.1 ± 102.9	2-24	+ 181	<0.01


* Pharmaco-invasive strategy refers to angiography or PCI in a period of 2-24 hours; in the present study, no patient met this criterion. STEMI: ST-elevation myocardial infarction, ACS-Gt: Acute coronary syndrome-Guatemala, ESC: European society of cardiology, ECG: electrocardiogram, PCI: percutaneous coronary intervention.

Results regarding healthcare system structure (Table 6) demonstrated that only 6.4% of ACS management was dictated by a cardiologist and the majority by an internal medicine resident. Quantitative and qualitative troponin was available in 81% and 9.2% of cases respectively. The National Cardiovascular Surgery Unit of Guatemala (UNICAR), a semi-public institution, was the only centre that performed elective coronary angiography on those referred by National Health System centres. Patients discharged with an appointment to UNICAR (whenever the patient wasn’t transferred during hospital stay, needed a second catheterization or coronary artery bypass graft) was around 10% in both groups. At discharge, the median time for a follow-up appointment was 20 days in STEMI and 7.5 days in NSTEMI/UA.

**Table 6:** Structure in the attention of the acute coronary syndrome.


	STEMI	NSTEMI/UA	TOTAL	P

	N = 88	N = 21	N = 109	

	N (%)	N (%)	N (%)	

**Physician responsible**				0.42

Internal medicine/emergency medicine resident	61 (69.3)	14 (66.7)	75 (68.8)	

Internist	13 (14.8)	6 (28.6)	19 (17.4)	

Cardiologist	6 (6.8)	1 (4.8)	7 (6.4)	

General physician	6 (6.8)	0.0	6 (5.5)	

Other	2 (2.3)	0.0	2 (1.8)	

**Availability**				

Fully working ambulance	87 (100)	21 (100)	108 (100)	–

Electrocardiogram	87 (98.9)	21 (100)	108 (99.1)	0.42

Hospital beds	87 (98.9)	21 (100)	108 (99.1)	0.64

Electrocardiogram paper	86 (97.7)	21 (100)	107 (98.2)	0.62

Any cardiac enzyme	80 (90.9)	15 (71.4)	95 (87.2)	0.01

Quantitative troponin	74 (84.1)	15 (71.4)	89 (81.7)	0.17

CKMB	73 (83)	12 (57.1)	85 (78)	0.01

Qualitative troponin	10 (11.4)	0.0	10 (9.2)	0.1

Any Fibrinolytic*	87 (98.9)	–	–	–

Streptokinase	86 (98.9)	–	–	–

Alteplase	63 (72.4)	–	–	–

**Discharged with appointment (UNICAR)**	8 (9.2)	2 (10)	10 (9.3)	0.78

	**MEDIAN (25–75)**	**MEDIAN (25–75)**	**MEDIAN (25–75)**	

Days until appointment	20.5 (11.7–27)	7.5 (7–8)	17.7 (8–24)	0.05


*During the data collection period, only streptokinase and alteplase was available in the National Ministry of Public Health network. STEMI: ST-elevation myocardial infarction, NSTEMI: non-ST elevation myocardial infarction, UA: unstable angina, CKMB: creatin kinase-MB, UNICAR: *“Unidad Nacional de Cirugía Cardiovascular*” (Cardiovascular surgery unit of Guatemala).

Table 7 analyses in-hospital medications in regards to OMT. Aspirin was given to the majority of patients (97%) with OMT of 80% and no difference between STEMI and NSTEMI/UA. Clopidogrel was given to 95%, with 58% OMT and was found significantly lower in NSTEMI/UA. Enoxaparin was the main anticoagulation treatment used (78%), and nearly half was optimally prescribed. Fibrinolytic therapy was administered to 59% of STEMI patients and 82% was optimal. One patient with NSTEMI/UA received fibrinolysis as a medical error (Supplement Table 3). Optimal in-hospital medical therapy was 8.3%, being less frequent in STEMI patients (6.8%) versus NSTEMI/UA (14.3%).

**Table 7:** In-Hospital Medical Therapy.


MEDICATION	PRESCRIPTION				OPTIMAL MEDICAL THERAPY			
	
	STEMI	NSTEMI/UA	TOTAL	P	STEMI	NSTEMI/UA	TOTAL	P
	
	N = 88	N = 21	N = 109		N = 88	N = 21	N = 109	
	
	N (%)	N (%)	N (%)		N (%)	N (%)	N (%)	

Aspirin	82 (95)	21 (100)	103 (97)	0.38	68 (83)	14 (67)	82 (80)	0.9

Clopidogrel	80 (94)	21 (100)	101 (95)	0.25	52 (66)	6 (29)	58 (58)	<0.01

Enoxaparin	18 (85)	18 (85)	83 (78)	0.35	31 (47)	9 (50)	40 (48)	0.86

Unfractionated Heparin	7 (82)	0.0	7 (6.6)	0.17	2 (28)	–	–	–

Fibrinolytic	52 (59)	–	–	–	41 (82)	–	–	–

**Optimal In-Hospital Medical Therapy**					6 (6.8)	3 (14.3)	9 (8.3)	0.26


STEMI: ST-elevation myocardial infarction, NSTEMI: non-ST elevation myocardial infarction, UA: unstable angina.

DOMT is described in Table 8. More than 70% and 92% of patients with rLVEF received aspirin and P2Y12 inhibitors respectively with optimal dosage. The majority received an optimal dose of Statins. In this high-risk group of patients, ACE inhibitors/ARB were prescribed to 50%, β-blocker to 78%, and ARNI to 42%. Fewer patients with NSTEMI/UA received DOMT with ACEI/ARB or β-blocker. More than 90% of patients with preserved LVEF, were prescribed aspirin, P2Y12 inhibitors, and statins with DOMT. Discharge optimal medical therapy in rLVEF was 78% and in preserved LVEF 83%.

**Table 8:** Discharge Medical Therapy.


MEDICATION	DISCHARGE PRESCRIPTION – LVEF ≤40%				OPTIMAL MEDICAL THERAPY – LVEF ≤40%			
	
	STEMI	NSTEMI/UA	TOTAL	P	STEMI	NSTEMI/UA	TOTAL	P
	
	N = 11	N = 3	N = 14		N = 11	N = 3	N = 14	
	
	N (%)	N (%)	N (%)		N (%)	N (%)	N (%)	

P2Y12 inhibitor	11 (100)	2 (66)	13 (92)	0.04	11 (100)	2 (100)	13 (100)	–

Statin	11 (100)	3 (100)	14 (100)	–	10 (90)	2 (100)	12 (92)	0.65

Aspirin	8 (72)	2 (66)	10 (71)	0.83	8 (100)	2 (100)	10 (100)	–

β-blocker	9 (81)	2 (66)	11 (78)	0.57	6 (100)	0.0	6 (85)	<0.01

ARNI	4 (36)	2 (66)	6 (42)	0.34	4 (100)	2 (100)	6 (100)	–

ACE inhibitor/ARB	6 (54)	1 (33)	7 (50)	0.51	5 (83)	0.0	5 (83)	<0.01

**Discharged Optimal Medical Therapy**					9 (81)	2 (66)	11 (78)	0.57

**MEDICATION**	**DISCHARGE PRESCRIPTION – LVEF ≥40%**				**OPTIMAL MEDICAL THERAPY – LVEF ≥40%**			

	STEMI	NSTEMI/UA	TOTAL	P	STEMI	NSTEMI/UA	TOTAL	P
	
	N = 43	N = 5	N = 48		N = 43	N = 5	N = 48	
	
	N (%)	N (%)	N (%)		N (%)	N (%)	N (%)	

P2Y12 inhibitors	39 (95)	5 (100)	44 (95)	0.61	39 (100)	5 (100)	44 (100)	–

Aspirin	39 (95)	4 (80)	43 (93)	0.19	39 (100)	4 (100)	43 (100)	–

Statin	38 (92)	5 (100)	43 (93)	0.53	34 (82)	5 (100)	39 (84)	0.31

**Discharged Optimal Medical Therapy**					36 (83)	4 (80)	40 (83)	0.83


LVEF: left ventricular ejection fraction, STEMI: ST-elevation myocardial infarction, NSTEMI: non-ST elevation myocardial infarction, UA: unstable angina, OMT: optimal medical therapy.

To understand Guatemala’s health system structure, it is important to describe the source of medications prescribed at hospital admission. Drug’s availability varied depending on the hospital and geographical location. Three possible sources were found: public hospital, patient’s expense, or donation. ACEI/ARB, aspirin, P2Y12 inhibitors, enoxaparin were mostly provided by the public hospital, whereas Statins and β-blockers were more commonly available by donation or patient’s expense (Supplement Table 4).

**Figure 2:**
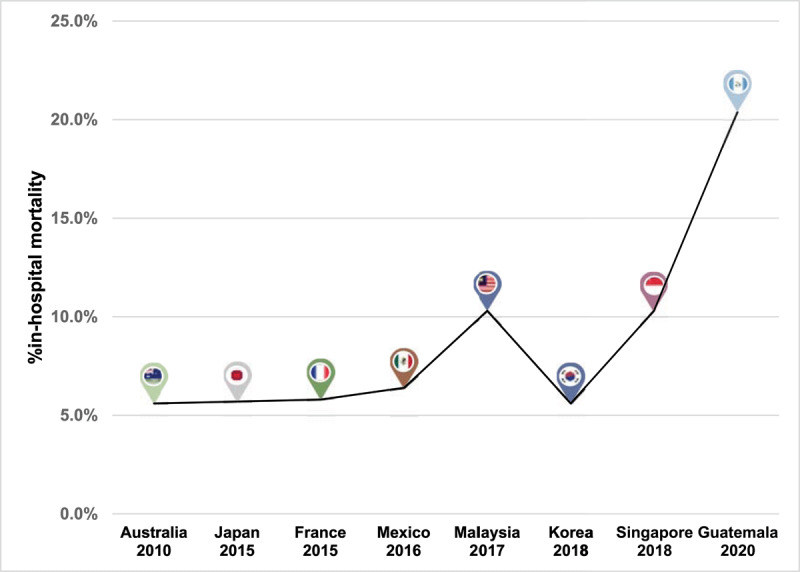
In-hospital ACS mortality [27,47,48].

In-hospital mortality was 20.4%, being non-significantly higher in NSTEMI/UA patients (Table 9, Figure 2) and considered among the highest currently reported. Cardiogenic shock was responsible for half of deaths. Mortality rate was analysed according to reperfusion status in STEMI patients; non-reperfused patients died twice as much as those successfully reperfused. Thirty-day mortality rate was 1.5% and 7.7% in STEMI and NSTEMI/UA respectively, nearly 5% of patients were lost during follow-up.

**Table 9:** Outcome.


	STEMI	NSTEMI/UA	TOTAL	P

	N = 88	N = 21	N = 109	

	N (%)	N (%)	N (%)	

**In-hospital mortality**	16 (18.4)	6 (28.6)	22 (20.4)	0.29

Cause				0.76

Cardiogenic shock	9 (56.3)	2 (33.3)	11 (50)	

Reinfarction	2 (12.5)	1 (16.7)	3 (13.6)	

Stroke	1 (6.3)	1 (16.7)	2 (9.1)	

Ventricular Arrythmias	1 (6.3)	0.0	1 (4.5)	

Other non-specified	3 (18.8)	2 (33.3)	5 (22.7)	

**In-hospital mortality and reperfusion status**				

Non-reperfused	9 (25.7)	–	–	–

Reperfused	7 (13.5)	–	–	–

**30-day mortality**				0.38

Lost in follow-up	3 (4.6)	1 (7.7)	4 (5.1)	

Deceased	1 (1.5)	1 (7.7)	2 (2.6)	

**In-hospital morbidity**	46 (52.9)	8 (38.1)	54 (50)	0.22

Cause				

Non-cardiovascular	35 (39.8)	8 (38.1)	43 (39.4)	0.88

Non-lethal ventricular arrythmia	4 (4.5)	1 (4.8)	5 (4.6)	0.96

Heart Failure	4 (4.5)	0.0	4 (3.7)	0.32

Non-lethal stroke	3 (3.4)	0.0	3 (2.8)	0.39

Haemorrhagic complication	3 (3.4)	0.0	3 (2.8)	0.39

Non-lethal reinfarction	3 (3.4)	0.0	3 (2.8)	0.39

Supraventricular Arrythmias	2 (2.3)	0.0	2 (1.8)	0.48

Reverted cardiac arrest	1 (1.1)	0.0	1 (0.9)	0.62

GUSTO classification				

Severe	1 (33.3)	–	–	–

Mild	2 (66.7)	–	–	–

Cardiogenic shock	16 (18.2)	2 (9.5)	18 (16.5)	0.33

Pharmacotherapy				0.79

Both	13 (81.3)	2 (100)	15 (83.3)	

Vasopressor	2 (12.5)	0.0	2 (11.1)	

Inotropic	1 (6.3)	0.0	1 (5.6)	

**Echocardiogram previous to discharge**	56 (63.6)	7 (33.3)	63 (57.8)	0.01

**Performed by**				

Private clinic	52 (89.7)	7 (87.5)	59 (89.4)	

National Ministry of Public Health	6 (10.3)	1 (12.5)	7 (10.6)	

	X̄ ± SD	X̄ ± SD	X̄ ± SD	

LVEF ∼ %	49.5 ± 9.3	42.1 ± 16.8	48.5 ± 10.6	0.06

	**median (25–75)**	**median (25–75)**	**median (25–75)**	

**In-hospital length of stay** ∼ **days**	10 (6–14)	7 (4.5–9)	8 (4–14)	0.45


STEMI: ST-elevation myocardial infarction, NSTEMI: non-ST elevation myocardial infarction, UA: unstable angina, LVEF: left ventricular ejection fraction.

More patients with STEMI suffered any type of in-hospital cardiovascular complication than NSTEMI/UA, of these, cardiogenic shock and non-lethal ventricular arrhythmias were more common. Around half of patients had an echocardiogram performed previous to discharge, most at a private hospital. Mean LVEF was 49% in STEMI patients and 42% in NSTEMI/UA (p = 0.06). Median in-hospital length of stay was 8 days, longer in STEMI patients.

During review of every electronic case, we found particular situations worth mentioning. Supplement Table 3 describes these erroneous diagnoses and management in detail.

## Discussion

Executing an ACS national registry fulfils recommendations of clinical performance and quality improvement of STEMI/NSTEMI measures from 2017 AHA/ACC [17]. A mixture of factors determines patient outcome after an ACS. These were explored and divided into categories corresponding to: health system process, structure, and outcome. Currently, there is no standard of care for STEMI nor NSTEMI/UA in Guatemala. This must be taken into consideration when reviewing the data collected.

Current evidence reports that NSTEMI/UA incidence has increased with respect to STEMI [18]. This contrasts with this registry’s data, where NSTEMI/UA represents 20% of patients. The rise in incidence is due to advances in diagnostic methods such as high–sensitivity troponin [19]. However, this diagnostic tool is not available in Guatemala’s public Health System.

The epidemiological profile found denotes the country’s reality and demonstrates patient vulnerability. Evidence shows that social factors play a major role in determining outcome after a cardiovascular event [20]. In the CREATE registry, worse outcome after an ACS was associated with lower socioeconomic status [21]. Furthermore, underdiagnosis of cardiovascular disease is more frequent among the poor, and they are less likely to receive evidence-based treatment due to necessary out-of-pocket expense [22,23].

ACS risk factors are similar to what has been described [3], however there is a rise in the prevalence of hypertension and diabetes. Tobacco use increased in incidence in patients with ACS. Guatemala implemented a smoke-free law, yet, its long-term compliance has decreased [24]. Although risk factors between both groups showed no difference, patients with NSTEMI/UA had a higher incidence of hypertension and diabetes mellitus, similar to the GRACE registry [25]. The INTERHEART Latin American Study reported that history of hypertension, diabetes mellitus, or current smoking were associated with higher risk of AMI [26].

In STEMI, fibrinolysis rate was 59% and no primary PCI was performed. This is mainly driven by late presentation (Table 2). Previous data reported a reperfusion rate of 28% in Guatemala [3]. Although this study was set during a different time frame and direct comparison is not possible, this registry shows an improvement in reperfusion rate. It was similar in comparison with other registries [27,28,29] and lower than Europe in 2004 (64%) [30].

Current European STEMI guidelines recommend use of fibrin-specific thrombolytic based on two trials [4,31,32]. However, recent evidence demonstrates that when streptokinase is administered and angiography is performed within 24 hours, coronary artery patency is 86%. This determines the importance of routine angiography [33]. Current guidelines on treatment delay use tenecteplase as the drug of choice for reperfusion, but it is not available in Guatemala’s Public Health System.

There is evidence that stable, non-reperfused patients do not benefit from immediate PCI when the artery is occluded [34]. Thus, non-invasive methods can be performed searching for ischaemia or viability. In this registry, the rate of non-reperfused STEMI patients was 41%. Around 20% were taken to angiography or PCI, and about half of them had a non-invasive test performed (dobutamine echocardiogram is the only method available). It is strongly recommended to perform PCI in patients with successful fibrinolysis between 2 to 24 hours [4,14]. No patients were taken to PCI within this interval, and only half were taken to angiography or PCI after 24 hours. When fibrinolysis was unsuccessful, there was no opportunity to perform rescue PCI. This delay is due to administrative and economic barriers.

In NSTEMI/UA, ischaemic and haemorrhagic risk assessments are key to decide when to perform coronary angiography. One-quarter of NSTEMI/UA patients had angiography (80%) or PCI (20%) performed within 6.4 days, despite high GRACE (mean 129 ± 32) and CRUSADE (mean 35.8 ± 20.8) scores (Tables 2 and 3). Coronary angiography rate is extremely low compared to other registries [25,30,35]. Clinical presentation (Table 3) demonstrated higher GRACE score in STEMI and more likely to have a TIMI score ≥7. Most patients in both groups had a Killip-Kimball I clinical presentation which has been described in other registries [21,30,36].

Only 22% of patients used emergency medical systems (EMS) (firefighters are the only available public EMS). Despite the fact that these are a critical component of the STEMI chain of survival [37]. Other studies found that low EMS use was related to prolonged treatment time, and could adversely affect patient prognosis [38]. Ambulance delays were prolonged in NSTEMI/UA patients, while FMC was prolonged in STEMI patients. In Brazil, median time from ambulance call to arrival improved after implementing a regional STEMI protocol [39].

In this cohort, FMC was 180 minutes (300 in STEMI vs. 185 in NSTEMI/UA). FMC was shorter in other series such as the STREAM trial [40]. Regional differences have been found. In Mexico, FMC was 120 minutes in patients receiving pharmacoinvasive strategy and 150 minutes in pPCI [41]. Guatemalan STEMI patients took twice as long to be diagnosed in comparison with Mexico and four times compared to Canada. Pre-hospital delay has been constantly reported high over a 20-year observational period in developed countries [42]. The characteristics of the EMS ambulances were suboptimal in comparison with other EMS [43]. All time intervals were significantly prolonged and these have proven to be prognostic indicators in STEMI [4].

Most patients were evaluated and cared by internal medicine residents (Table 6). Current evidence shows that patients admitted to non-cardiology services received fewer secondary prevention medications and had worse outcomes [44]. At the time of ACS diagnosis, basic resources for establishing diagnosis and lytic therapy were available. However, their use was not always adequate (supplement Table 3).

Pharmacological secondary prevention of ACS is an important factor that improves patient outcome and should be dose adjusted according to clinical condition (age, loading dose, renal function, etc.) (Table 7) [4,12]. Although initial pharmacological treatment was widely prescribed (acetylsalicylic acid, P2Y12 inhibitors, heparins, and fibrinolytic) in both STEMI and NSTEMI/UA patients, OMT was extremely low (8.3%). In a registry performed in patients with similar socioeconomic characteristics, those receiving optimal in-hospital medical therapy were not associated with in-hospital death nor major adverse cardiovascular events [45]. It is worth mentioning that in Guatemala, a drug is not always provided by the National Ministry of Public Health (supplement Table 2). This may partially explain the low rate of OMT. Discharge OMT was analysed in patients with and without rLVEF (Table 8). Patients with rLVEF and NSTEMI/UA received less OMT than their counterparts, similar findings have been described [46].

**Figure 3:**
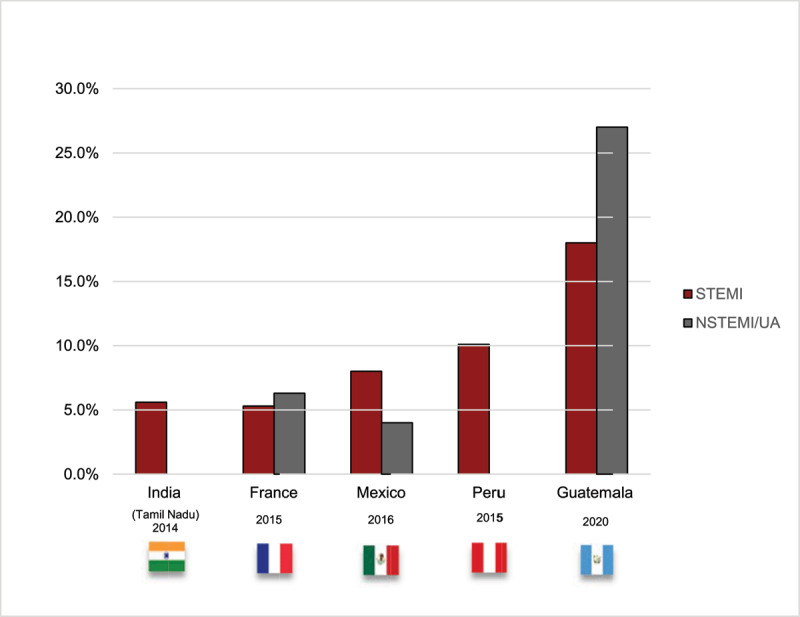
Mortality by type of ACS. STEMI: ST-elevation myocardial infarction, NSTEMI: non-ST elevation myocardial infarction [27,28,47,49].

Outcomes are shown in Table 9. In-hospital mortality of ACS was 20.4%, it is among the highest mortality reported (Figure 2) [21,27,47,48]. When evaluating mortality by type of ACS it remained substantially high in comparison with other registries (Figure 3) [27,28,47,49]. In STEMI, mortality rate was 13.5% and 25.7% in reperfused and non-reperfused respectively. Similar evidence described that non-reperfused patients have higher adjusted 30-day mortality [50].

Half of patients presented at least one cardiovascular in-hospital morbidity, cardiogenic shock being the most frequent in STEMI and NSTEMI/UA. Other data reports fewer prevalence of cardiogenic shock in STEMI patients [28], although it is still the most common complication.

European STEMI and NSTEMI guidelines determine routine echocardiography to be a recommendation IB and IC respectively [4,12]. This registry showed that 63% of STEMI patients and 33% of NSTEMI/UA patients had an echocardiogram performed before discharge.

In-hospital length of stay was 8 days. Prolonged in-hospital length of stay may be secondary to lack of PCI and echocardiogram at Public Hospitals, high prevalence of in-hospital cardiovascular complications, and administrative delays. Recommendations exist about the safe length of stay in patients with STEMI, however, they do not apply since these refer to patients with complete revascularization and clinical stability.

The metrics of a weak health system were described in detail and associated with a high burden of mortality secondary to ACS. This is the first high-quality data registry in Guatemala that describes the specific problems of the process and structure of ACS.

## Study Limitation

This registry provided data exclusively from the public health system, limiting the power of statistical findings. Future registries should asses private, and semi-public health services.

## Conclusion

Guatemala is a middle-income country without real-world data on ACS. This is the first cohort of Guatemala’s National Acute Coronary Syndrome Registry representing 6 out of the country’s 8 geographical regions. A vulnerable epidemiologic profile was found, characterized by high burden, non-communicable disease, low educational level, high rates of unemployment, and poor access to guide in-hospital directed medical therapy for STEMI and NTSTEMI/UA.

In STEMI, delays are translated to worse patient outcome. This study found prolonged first medical contact, total ischaemic time, and lack of fulfilment of the recommended time period between successful fibrinolysis and PCI. ACS management was mostly dictated by general physicians. Even though the majority of Public Hospitals had access to the necessary resources for diagnosis and treatment of ACS, unacceptable outcomes were described (in-hospital mortality rate of 20%). Cardiogenic shock was the leading cause of in-hospital mortality and morbidity. There was a low rate of echocardiograms performed on patients independent of ACS type.

Out-of-pocket patient expense was considerable in regards to ambulance transfer to a referral hospital (after ACS diagnosis), in-hospital medication, and essential medical workup such as echocardiogram.

This registry could contribute to the understanding of ACS as the main cause of death in Guatemala, and how resources that are already limited are inefficiently used. It could become an important reference in order to justify the imperative need for a restructuration of the healthcare system structure and policy.

## Data Accessibility Statement

The data supporting the findings of this study are available within this article and its supplementary materials.

## Additional File

The additional file for this article can be found as follows:

10.5334/gh.1168.s1 Supplementary Tables. Tables 1 to 4.
