# Supplementary material for: Structure, Process, and Mortality Associated with Acute Coronary Syndrome Management in Guatemala’s National Healthcare System: The ACS-GT Registry
Source: Glob Heart. 2022 Dec 1;17(1):84. doi: 10.5334/gh.1168 (PMC9717345; doi:10.5334/gh.1168)
Supplement: Supplementary Tables. — Tables 1 to 4. [file gh-17-1-1168-s1.pdf]

Supplement Table 1. Variable definitions

Variable Definition

AMI

The evidence of myocardial injury (elevation of cardiac troponin values with at least one result above the 99th percentile upper reference limit) in a clinical setting consistent with myocardial ischemia (1).

ACS

The presence of any of the following: ST-segment elevation myocardial infarction (STEMI), non-ST-segment elevation myocardial infarction (NSTEMI) and unstable angina (UA) (1).

STEMI

The presence of persistent chest discomfort or other symptoms suggestive of ischemia and ST-segment elevation in at least two contiguous leads (≥2.5mm in men <40years, ≥2mm in men ≥40years, ≥1.5mm in women in leads V2-V3 and/or ≥1mm in the other leads, or ≥0.5mm in leads V7-V9). The presence of a right bundle branch block with ST-segment elevation or left bundle branch block that met Sgarbossa criteria (1)

NSTEMI

Persistent chest discomfort without ST-segment elevation in ECG, but with troponin elevation, that may include transient ST-segment elevation, persistent or transient ST-segment depression, T-wave inversion, flat T waves, or pseudo normalization of T waves (2).

UA

The presence of chest discomfort without troponin elevation in the presence or absence of ischemic changes on the electrocardiogram (2).

Risk factors

Systemic
hypertension

Previous diagnosis or extremely high blood pressure at hospital arrival (Stage III >180/110) with or without additional evidence of organ damage (3).

Diabetes Mellitus

Diabetes mellitus was considered with history of anti-diabetic medication or typical hyperglycaemic symptoms, random blood glucose concentration ≥200 mg/dl, HbA1c ≥6.5%, or symptoms and laboratory findings suggestive of diabetic ketoacidosis or hyperosmolar hyperglycaemic state (4).

Measures to evaluate the process of care in acute coronary syndrome

First Medical Contact

The period of time between the beginning of any symptoms suggestive of ischemia and the evaluation by physician, paramedic, nurse or other who can obtain and interpret an electrocardiogram (1).

Door-to-needle time

The duration between patients’ arrival to a fibrinolytic capable centre and the start of fibrinolysis (1).

Door-to-balloon time

The time interval between the patient´s arrival to a PCI capable centre and the time of the first balloon inflation (for STEMI) (1).

Door-in to door-out
time

The duration between arrival of the patient at a non-capable PCI centre to discharge of the patient in an ambulance on the way to a PCI centre (1).

Total ischaemic time

The total time between the first symptom compatible with ACS and successful reperfusion therapy (pharmacologic or invasive) (1).

Reperfusion therapy

Failed fibrinolysis

Measured 90 minutes after the end of the fibrinolysis, the presence of any of both: ST-segment resolution <50% or hemodynamic or electrical instability, worsening ischemia, or persistent chest pain (1).

pPCI

Emergent PCI with balloon, stent, or other approved device, performed on the infarct related artery without previous fibrinolytic treatment (1).
Rescue angioplasty Emergent PCI performed as soon as possible in the case of failed fibrinolytic treatment (1).
Routine early PCI
strategy after
fibrinolysis,
Coronary angiography, with PCI of the infarct related artery, performed between 2 and 24 hours after successful fibrinolysis (1).
Supplementary Files
Pharmacoinvasive
strategy
Fibrinolysis combined with rescue PCI (in case of failed fibrinolysis) or routine early PCI strategy (in case of successful fibrinolysis) (1).

Mortality and morbidity causes

Cardiogenic shock
Cardiogenic shock is defined by:

- systolic blood pressure <90 mmHg without the support of vasoactive substances, or 100 mmHg with vasopressors;
- clinical, chest X-ray expression of pulmonary capillary hypertension, or both;
- signs of peripheral vascular hypoperfusion manifested by oliguria <1 ml/kg/h;
- metabolic acidosis;
- cardiac index <2.2 l/min/m2;
- pulmonary capillary pressure >18 mmHg;
- arteriovenous oxygen difference >5.5 mL/dL. (5)

Haemorrhagic
complication
Non-fatal bleeding associated with the use of fibrinolytics, aspirin, P2Y12 inhibitors, heparin (UFH, LWMH), GpIIb/IIIa inhibitors, or oral anticoagulants (5).
GUSTO Bleeding Classification was used as follows:

- Severe: Either intracranial haemorrhage or bleeding that causes hemodynamic compromise and requires intervention.
- Moderate: Bleeding that requires blood transfusion but does not result in hemodynamic compromise.
- Mild: bleeding that does not meet the criteria for either severe or moderate bleeding.

AMI: acute myocardial infarction, ACS: acute coronary syndrome, STEMI: ST-elevation myocardial infarction, NSTEMI: non-ST elevation myocardial infarction, UA: unstable angina, PCI: percutaneous coronary intervention, pPCI: primary percutaneous coronary intervention.

Supplement Table 2. Biochemical characteristics at admission
STEMI NSTEMI/UA
n=88 n=21 p
median (25-75) median (25-75)
NT pro-BNP ~pg/mL 1454 (328.2–2602) 5725.5 (1059.2–8975.7) 0.16
Glucose ~mg/dL 148 (116–296.2) 126 (110.5–214) 0.33
CKMB ~ U/L 44 (27–100.6) 88.1 (48.2–214.4) 0.72
Creatinine ~ mg/dL 1.03 (0.76–1.50) 0.93 (0.76–1.29) 0.34
X±DE X±DE
Trop T~ ng/mL 1717.5±2540.6 963.1±1111.8 0.46
LDL~mg/dL 102.8±40.7 95.7±43.2 0.67
HDL~mg/dL 35.2±9.6 36.3±11.27 0.78
Hb~g/dL 14.3±2.3 13.5±2.31 0.14
Uric acid~mg/dL 6.5±2.3 5.9±2.6 0.56

Trop I~ng/mL	38.2±170.1	5.8±5.5	0.6

STEMI: ST-elevation myocardial infarction, NSTEMI: non-ST elevation myocardial infarction, UA: unstable angina, CKMB: creatinine kinase-MB, NT pro-BNP: N-terminal pro-hormone BNP, Trop: troponin, LDL: low density lipoprotein, HDL: high density lipoprotein, Hb: haemoglobin.

Supplement Table 3. Extraordinary circumstances

	**Total n=109 n (%)**
Erroneous NSTEMI diagnosis (ECG evidence of STEMI)	7 (6.4)
Not reperfused within treatment window (FMC <12 hours)	4 (3.66)
Erroneous STEMI diagnosis (ECG without evidence of STEMI)	2 (1.8)
Fibrinolytic administered erroneously (FMC >12 hours and clinically stable STEMI)	2 (1.8)
Fibrinolytic administered in NSTEMI/UA	1 (0.91)
Self-reperfused STEMI by ECG (in correlation to FMC and troponin)	1 (0.91)

STEMI: ST-elevation myocardial infarction, NSTEMI: non-ST elevation myocardial infarction, UA: unstable angina, ECG: electrocardiogram, FMC: first medical contact.

Supplement Table 4. Source of medication administered at admission.

	**STEMI n=88 n (%)**	**NSTEMI/UA n=21 n (%)**	**Total n=109 n (%)**	**p**
**ACEI/ARB**				0.9
Public hospital (MSPAS)	14 (82.4)	4 (80)	18 (82)	
Patient	3 (17.6)	1 (20)	4 (18)	
**Statins**				0.15
Donation	47 (56.6)	11 (52.4)	58 (55.8)	
Public hospital (MSPAS)	17 (20.5)	8 (38.1)	25 (24)	
Patient	19 (22.9)	2 (9.5)	21 (20.2)	
**Beta-blockers**				0.53
Patient	6 (66.7)	2 (50)	8 (61.5)	
Public hospital (MSPAS)	2 (22.2)	2 (50)	4 (30.8)	
Donation	1 (11.1)	0	1 (7.7)	
**Acetylsalicylic acid**				0.47
Public hospital (MSPAS)	74 (90.2)	20 (95.2)	94 (91.3)	
Patient	8 (9.8)	1 (4.8)	9 (8.7)	
**P2Y12**				0.38
Public hospital (MSPAS)	55 (68.8)	17 (81)	72 (71.3)	
Patient	12 (15)	3 (14.3)	15 (14.9)	
Donation	13 (16.3)	1 (4.8)	14 (13.9)	
**Unfractionated heparin**				-
Public hospital (MSPAS)	7 (100)	-	-	
**Enoxaparin**				0.54
Public hospital (MSPAS)	54 (83.1)	16 (88.9)	70 (84.3)	
Patient	11 (16.9)	2 (11.1)	13 (15.7)	
**Second dose of Enoxaparin**				0.31
Public hospital (MSPAS)	27 (79.4)	4 (100)	31 (81.6)	
Patient	7 (20.6)	0	7 (18.4)	
**Nitrates**				0.54
Public hospital (MSPAS)	3 (75)	1 (50)	4 (66.7)	
Patient	1 (25)	1 (50)	2 (33.3)	

STEMI: ST-elevation myocardial infarction, NSTEMI: non-ST elevation myocardial infarction, UA: unstable angina, ACEI: Angiotensin-converting enzyme inhibitors, ARB: Angiotensin II receptor blocker, MSPAS: "*Ministerio de Salud Pública y Asistencia Social*" (National Ministry of Public Health).

REFERENCES

1. Ibanez B, James S, Agewall S, et al. 2017 ESC Guidelines for the management of acute myocardial infarction in patients presenting with ST-segment elevation. European Heart Journal. 2017;39(2):119-177. DOI: 10.1093/eurheartj/ehx393
2. Roffi M, Patrono C, Caollet JP, et al. 2015 ESC Guidelines for the management of acute coronary syndromes in patients presenting without persistent ST-segment elevation. European Heart Journal. 2016;37(3):267-315. DOI: 10.1093/eurheartj/ehv320
3. Williams, B, Mancia G, Spiering W, et al. 2018 ESC/ESH Guidelines for the management of arterial hypertension. European Heart Journal. 2018; 39(33): 3021-3104. DOI: 10.1093/eurheartj/ehy339
4. American Diabetes Association. Classification and diagnosis of diabetes: Standards of medical care in diabetes-2020. Diabetes Care. 2020; 43(1): 14-31. DOI: https://doi.org/10.2337/dc20-S002
5. Cannon CP, Brindis RG, Chaitman BR, et al. 2013 ACCF/AHA Key Data Elements and Definitions for Measuring the Clinical Management and Outcomes of Patients with Acute Coronary Syndromes and Coronary Artery Disease. Circulation. 2013; 127:1052-1089. DOI: https://doi.org/10.1161/CIR.0b013e3182831a11
